# Improving frequency and content of referral correspondence between general practitioners and psychiatrists: a cross-sectional descriptive study

**DOI:** 10.1017/S1463423625100492

**Published:** 2025-10-24

**Authors:** Céline Bouton, Anne-Victoire Fayolle, Eric Cailliez, Aline Ramond, Clément Guineberteau

**Affiliations:** 1 Univ Angers, POPS, SFR ICAT, F-49000 Angers, France; 2 Department of General Medicine, Faculty of Health, Nantes University, 44000 Nantes, France; 3 Department of General Medicine, Faculty of Health, University of Angers, 49000 Angers, France

**Keywords:** Correspondence as topic, General Practitioners, mental health, Primary health care, Psychiatrists

## Abstract

**Background:**

Written referrals and follow-up correspondence between referring general practitioners (GPs) and psychiatrists is a medico-legal responsibility and integral part of caring for patients with mental illness. Objective: To describe expectations and practices that GPs and psychiatrists have when exchanging correspondence concerning patients with mental health problems.

**Methods:**

In this observational, declarative, and cross-sectional study, two surveys were used to evaluate the expectations, frequency, and content of correspondence exchanged between GPs and psychiatrists. The questionnaire was based on the National College for the Quality of Psychiatric Care 2010 recommendations. Conducted in a regional setting in France between 2014 and 2016, the study involved 2754 GPs and 575 psychiatrists.

**Results:**

Overall, we achieved a positive response rate of 33% (189/575) of psychiatrists and 23% (628/2754) of GPs, which was similar in each local region. Regarding the correspondence from GPs to psychiatrists, 478 (75%) GPs declared having written a referral for an initial consultation and 84 (44%) psychiatrists declared having received a referral. Regarding the correspondence from psychiatrists to GPs, 144 (76%) psychiatrists declared having written at least one letter after the initial consultation or during follow-up and 160 (25%) GPs declared having received return correspondence. The GPs would like to be better informed about psychotherapeutic or long-term management, leave of absence from work, surveillance measures, prognosis, and division of specialist roles. The psychiatrists would like to receive more information about previous medication trialed, the level of willingness the patient has to consult a psychiatrist and any allergies or intolerances to medication.

**Conclusion:**

This study highlights the need to improve the disparity between expected and received correspondence from GPs and psychiatrists concerning patients with mental health problems.

## Background

Written referrals and clear, prompt follow-up correspondence between referring general practitioners (GPs) and psychiatrists is a medico-legal responsibility and integral part of caring for patients with mental illness (French National Authority for Health 2018a; Gallais 2014; Légifrance 2023; Ministry of Health and Access to Care 2021; WoNCa Europe 2023 Edition 2023). Importantly, effective inter-disciplinary correspondence confirms medical prescriptions and limits care delays and interruptions in this vulnerable population. These delays can cause a combination of premature mortality, handicap, severe disabilities, and social exclusion (Byrne 2023; Edmunds 2018; John *et al.* 2018; Roberts *et al.* 2017).

In France, GPs are the first contact point for a large proportion of patients with mental health problems, highlighting their frontline role in managing mental healthcare in the community. Additionally, 7 out of 10 GPs report seeing more frequent requests for mental health care since the start of the COVID pandemic (Bergeat *et al.* 2021; Byrne *et al.* 2021).

Already, in 2010 good practice guidelines published by the National College for the Quality of Psychiatric Care (CNQSP) suggest GPs and psychiatrists should share useful information including physical and psychological dimensions, patient preferences and respect patient rights and autonomy. This information should be included in referral letters from the GP and follow-up correspondence from the psychiatrist (Cohen *et al.* 2021). To improve the diagnosis and management of patients with mental health disorders, the French National Authority for Health (Haute Autorité de Santé, HAS) published a good practice recommendation in 2011, updated in 2018, which includes a specific framework for coordination and communication between GPs and mental health professionals (French National Authority for Health 2011, 2018b). These guidelines stipulate that GPs may manage psychological problems, implement further screening tests or the post-discharge period when a mental health problem is managed with a psychiatric team. There are few recent studies in the literature that have specifically examined the correspondence between GPs and psychiatrists (Cohen *et al.* 2021; Kinouani *et al.* 2024).

Effective mental healthcare management is particularly important in France, which has the highest number of mental health illness in Europe. In France, one out of five people is affected by psychological distress and 23 million euros is spent each year on psychotropic drugs to manage mental health problems, despite the controversy concerning the efficacy of these medicines without a psychiatric diagnosis (GBD 2019 Mental Disorders Collaborators 2022; Ministry of Health and Access to Care 2021; Ministry of Labour, Health, Solidarity, and Families 2023).

However, GPs have previously reported facing a lack of psychiatrist feedback as a major difficulty when fulfilling their central role in organizing mental health care (Linder *et al.* 2019).

## Methods

### Aim

The aim of this research was to describe the current frequency and content of GPs and psychiatrists correspondences in outpatient treatment of adult patients with mental health problems in France.

### Design

This observational, declarative, descriptive and analytical study was conducted in parallel in five areas in the Pays-de-la-Loire region, France between 2014 and 2016.

### Population studied

Full-time, primary care GPs registered with the French national medical association (Ordre des médecins), having an established general practice and managing patients with mental health problems in the community were included. GPs who were not contactable by email were excluded. Consulting psychiatrists were included but paediatric psychiatrists were excluded.

### Collecting data

One academic GP and two psychiatrists designed two distinct questionnaires, one for the GPs and one for psychiatrists (See questionnaires in supplementary material). The questions were based on the CNQSP recommendations on the methods of correspondence between GPs and psychiatrists and the researchers own experiences (French National Authority for Health 2011). The questionnaire for the GPs contained 45 questions and the psychiatrists contained 44 questions. Each questionnaire contained three sections. The first concerned the socio-demographic criteria, the second explored correspondences sent and received (frequency and content), and the third the preferred modes of correspondence. The interest of GPs in treating mental illnesses and psychological distress was evaluated using a Likert scale graduating from 1 to 10. Although the two questionnaires were independent, to remain pragmatic and clear, the main results concerning frequency and content will be presented with first the correspondence from GPs to psychiatrists followed by the correspondence from psychiatrists to GPs. The symbols * and ** will indicate the source of the responses: * for data from the GPs’ questionnaire and ** for data from the psychiatrists’ questionnaire. GPs and psychiatrists received the questionnaire via email via Sphinx®Online or letter and data were collected using Epi Info (version 7). All participants gave their consent to participate in this study and responded anonymously.

### Analysis

Quantitative variables are represented as absolute number (percentage), unless otherwise stated. In the analysis, the responses ‘always’ and ‘often’ were grouped together with ‘yes’ and ‘rarely’ and ‘never’ were grouped with ‘no’.

Variables with missing data were coded with an ‘unknown’ category.

## Results

*Among the 2,754 GPs solicited for the study 628 (22.8%) responded and are described in Table 1. Notably, 101 (16.1%) had had further training in treating psychological distress. Most GPs 558 (88.9%) were interested in treating mental health-related problems and psychological distress (Likert Scale rating ≥ 6) and two-thirds 389 (61.9%) were very interested, Likert Scale rating ≥ 8).

**Table 1.** Characteristics of participating general practitioners and psychiatrists

	General practitioners* *n* = 628	Psychiatrists** *n* = 189
**Sex (Female)**	281 (44.8%)	84 (44.4%)
**Age (Years)**		
<45	249 (39.7%)	57 (30.2%)
45–60	338 (53.8%)	88 (46.6%)
>61 years	39 (6.2%)	43 (22.8%)
**Place of work**		
Private outpatient practice only	575 (91.6%)	74 (39.2%)
Hospital practice (public or private)	0	94 (49.7%)
Hospital and outpatient practice	44 (7.0%)	20 (10.6%)

**Among the 575 psychiatrists solicited, 189 (33%) responded and are described in Table 1.

Among those who responded, 27 GPs* (4.3%) and 63 psychiatrists** (40.1%) were familiar with the CNQSP correspondence recommendations. Among these, 11 GPs* (40.7%) and 31 psychiatrists** (49.2%) considered the recommendations to be realistic.

### Correspondence from GP to psychiatrist

#### Frequency

*Concerning correspondence frequency, 476 (75.8%) GPs stated that they wrote a referral letter for the first psychiatrist consultation. There were 121 (19.3%) who wrote a follow-up letter.

**One hundred five psychiatrists (55.6%) declared not receiving a letter from GPs. One hundred fifty-five (82%) psychiatrists expected to receive a GP referral.

#### Content

Domains of correspondence content GPs communicated to psychiatrists included patient history, medical treatment, and clinical examination findings and are presented in Table 2.

**Table 2.** Domains of correspondence content from GPs to psychiatrists (*survey of GPs, **survey of psychiatrists)

Domains of correspondence content	Expected by psychiatrists** *n* = 189	Stated received by psychiatrists** *n* = 189	Stated sent by GPs* *n* = 628
**Patient history**			
Medical history	169 (89.4%)	106 (56.1%)	476 (75.8%)
Psychiatric history	164 (86.8%)	109 (57.7%)	514 (81.8%)
Life history	141 (74.6%)	73 (38.6%)	412 (65.6%)
**Patient medication**			
Actual psychoactive treatment	171 (90.5%)	145 (76.7%)	525 (83.6%)
Previous psychoactive treatment	156 (82.5%)	36 (19.0%)	459 (73.1%)
Drug intolerance	152 (80.4%)	37 (19.6%)	343 (54.6%)
Drug Allergy	129 (68.3%)	25 (13.2%)	313 (49.8%)
**Clinical examination**			
Initials symptoms	163 (86.2%)	136 (72.0%)	419 (66.7%)
Addictions	155 (82.0%)	105 (55.6%)	468 (74.5%)
Symptom evolution	149 (78.8%)	93 (49.2%)	372 (59.2%)
Actual management	142 (75.1%)	103 (54.5%)	470 (74.8%)
Acceptation of psychiatric consultation	117 (61.9%)	38 (20.1%)	187 (29.8%)

*One hundred thirty-five (21.5%) of GPs reported introducing that patient introduction solely to ensure social security reimbursement.

**Whilst, 88 (46.6%) psychiatrists, letters from GPs were just a letter of introduction for reimbursement as part of the care pathway.

**Six pieces of information psychiatrists require frequently were present in over 50% of referrals. This concerned treatment with psychoactive drugs (76.7% n = 145), symptoms identified (72.0% n = 136), psychiatric and physical history (57.7% n = 109 and 56.1% n = 106), addictions (55.6 n = 105), and actual management (54.5% n = 103). However, four pieces of information that psychiatrists particularly need were missing from more than 80% of letters. *Although GPs claimed to mention them in more than 50% of cases. These were the degree to which the patient accepted psychiatric evaluation, psychotropic treatments already tried, intolerance to drugs and allergies.

### Correspondence from psychiatrist to GP

#### Frequency

**One hundred forty-four (76.2%) respondent psychiatrists stated they write at least one letter during the follow-up period and 104 (55.0%) write a letter after the first consultation.

**Figure. 1.**
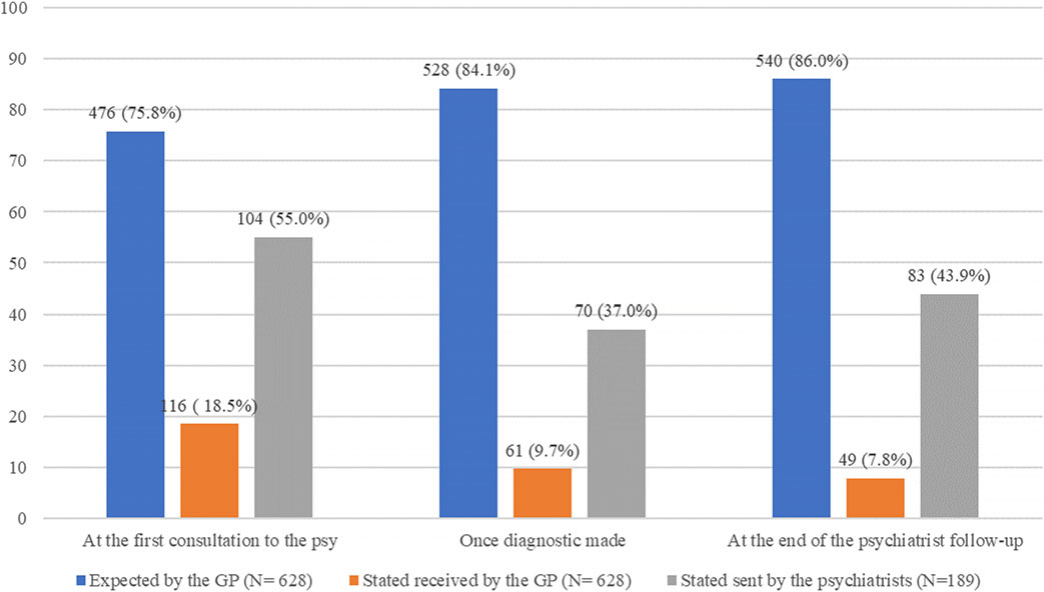
Frequencies of correspondence from psychiatrists to general practitioners. *Source*: GP, general practitioner.

*Ninety-three-point one per cent of the GPs (585/628) stated that correspondence was irregular with psychiatrists during patient follow-up. One hundred sixteen respondent GPs (18.5%) stated receiving a correspondence after the first psychiatrist consultation, 61 (9.7%) once the diagnosis was made, and 49 (7.8%) at the end of the psychiatric treatment (Figure 1). However, 25.5% (160/628) stated that they received at least one letter from the psychiatrist during the follow-up period.

#### Content

Domains of contents of correspondence from psychiatrists to GPs are presented in Table 3. *Content of the treatment prescribed by psychiatrists that GPs received and expected was found in 60.8% of correspondence (n = 382). Ten elements GPs need were present in less than 20% of psychiatrist correspondence: proposals and methods of psychotherapeutic treatment (14.8% n = 93), the necessity to classify a chronic illness (Affection de Longue Durée) (18.2% n = 114), the need for sick leave (16.4% n = 103), elements of biomedical and clinical monitoring (10.2% n = 64), risk of progression (10.5% n = 66), proposal for a care pathway (9.1% n = 57), proposal for social care (8.9% n = 56), and the sharing of roles (8% n = 50).

**Table 3.** Domains of correspondence content from psychiatrists to GPs (*survey of GPs, **survey of psychiatrists)

Domains of correspondence content	Expected by GPs* *n* = 628	Stated received by GPs* *n* = 628	Stated sent by psychiatrists** *n* = 189
**Diagnosis**			
Diagnostic hypothesis	575 (91.6%)	315 (50.2%)	171 (90.5%)
Progression risk	485 (77.2%)	66 (10.5%)	89 (47.1%)
Environmental factors	311 (49.5%)	155 (24.7%)	151 (79.9%)
**Treatment**			
Drug treatment	578 (92.0%)	382 (60.8%)	140 (74.1%)
Need for psychotherapeutic treatment	479 (76.3%)	120 (19.1%)	157 (83.1%)
Methods of psychotherapeutic treatments	455 (72.5%)	93 (14.8%)	144 (76.2%)
Biological and clinical monitoring	472 (75.2%)	64 (10.2%)	102 (54.0%)
Therapeutics propositions	551 (87.7%)	313 (49.8%)	164 (86.8%)
**Follow-up**			
Follow-up organization	527 (83.9%)	188 (29.9%)	176 (93.1%)
Role of the GP in follow-up	485 (77.2%)	50 (8.0%)	105 (55.6%)
**Administrative**			
Social care	387 (61.6%)	56 (8.9%)	312 (49.7%)
Need for sick leave	383 (61.0%)	103 (16.4%)	123 (65.1%)
Need to classify a chronic illness	437 (69.6%)	114 (18.2%)	131 (69.3%)

## Correspondence mode

GPs preferred using secure electronic messaging (50.4% n = 379) or traditional post (56.4% n = 354), than e-mail (21.7% n = 136), fax (10.8% n = 68) or by handing the letter over to the patient (18.2% n = 114).

## Discussion

### Main results

This is the first study to evaluate the difficulties and expectations from both GPs and psychiatrists concerning patient correspondence. This study supports previous research about the frequency and content of GP and psychiatrist correspondence and highlights considerable discrepancies between the professions. In general, both GPs and psychiatrists reported needing regular, detailed correspondence, in line with CNQSP recommendations, despite their lacking awareness about these recommendations. However, correspondence between GPs and psychiatrists were seen as being insufficient in both quantity and content. Furthermore, the declarations each profession made about the correspondence frequency varied remarkably from that actually received. Nevertheless, most GPs seemed interested in treating mental health, despite few having had further training in treating psychological distress.

Despite HAS recommendations encouraging frequent physician correspondence, only one quarter of GPs interviewed reported receiving a follow-up letter from a psychiatrist concerning their patient (French National Authority for Health 2018b). This corresponds with a small Californian study in which only 30 patients, (21%) had any indication from the mental health practitioner on the primary care record within three months of the initial consultation (Colaiaco *et al.* 2018). This lack of communication has been previously suggested to be due to fears of betraying patient confidentiality, or to respect medical and doctor-patient confidentiality (Mercier 2014). Also, whilst psychiatrists appreciate the GP’s proximity to the patient’s family as an information source, they fear disclosing information over which they want to retain control (Laboutière 2017).

It was reassuring to see that concerning correspondence content, GPs expected to receive a complete treatment and management report similar to reports in the UK and Australia (Selzer and Foley 2009; Serfontein *et al.* 2011). Unfortunately, psychiatrists often omitted to communicate critical elements such as: progression risk, psychotherapeutic treatment needs and methods, biological and clinical monitoring, chronic illness classification, sick leave prescribed, social care proposed, or role sharing. This lack of detail is concerning for GPs who expect the psychiatrist to confirm the diagnosis and so delay implementing treatment to avoid causing undue stress whilst the diagnosis remains uncertain (Gallais 2014). However, we found GPs are mostly concerned with psychiatrists putting a treatment into place (50%) than confirming a diagnosis (9.3%). Which is similar to reports in France for diagnosis confirmation which are lower for psychiatrists (7.1%) than other specialists (28%) (Passerieux and Younes 2007). Moreover, a separate study found that 83.7% of consultations were driven by therapeutic reasons, while only 35.9% were related to diagnostic concerns, highlighting the predominance of treatment-focused requests in that context (Kinouani *et al.* 2024).

Concerning GP correspondence content, most psychiatrists expected to receive a referral from the referring GP containing a maximum of information. However, psychiatrists often lacked receiving information about patient acceptance for psychiatric evaluation, previous psychotropic treatments tried, intolerance to drugs and allergies. Yet, GPs claimed to have mentioned them in more than 50% of cases. This confers with NHS (UK) findings in a project to standardize correspondence, in which referral letters lacked background health information, concerns of risk, and treatment tried (Odelola and Jabbar 2017). This lower level of detail may be due to a lack of GP training in psychiatric clinical reasoning, theory and practice. This means practitioners may write brief letters that have little psychiatric terminology. Also, GPs lack time to write detailed letter and are hindered by the time it takes for a letter to be delivered. They have been reported to prefer communication by phone. This allows certain problems to be resolved with a brief consultation (Hérisson 2015). Moreover, it has been found that GPs tend to report higher rates of feedback communications when they engage in more informal interactions with any specialists, suggesting that these informal exchanges might enhance communication despite the limited detail in written referrals (Scaioli *et al.* 2020). Yet written correspondence is required for medico-legal reasons.

According to psychiatrists, nearly half the GP referrals only wrote a short letter introducing the patient to ensure they would be reimbursed for psychiatric care. However, if referrals are brief and limited to this aim, there is little interdisciplinary exchange and may suggest that referring GP lack interest or competence concerning psychiatric disorders. This observation aligns with studies suggesting a lack of mental health training among GPs, which may contribute to their limited ability to provide comprehensive psychiatric referrals or to feel less confident in managing patients (Ayalon *et al.* 2016; Tzartzas *et al.* 2022).

Concerning the mode of correspondence, we found that most GPs were concerned about protecting patient confidentiality, preferring secure messaging platforms. However, psychiatrists have been found to be poorly informed and infrequently use encrypted messaging systems, even though it is secure and hastens information transfer (Hérisson 2015). Also, some GPs preferred sending a letter via the patient. However, in the French healthcare system this requires the patient to accept to be referred by their family doctor (Legifrance 2023). Whilst medical confidentiality is preserved, it may be problematic relying on patient cooperation as many patients are unaware of inter-professional communication practices. Alternatively, short phone calls from a psychiatrist to the GP following a psychiatric admission has been shown to improve signs and symptoms of depression (Burian *et al.* 2016).

Moving forward, any improvements in communication will require both GPs and psychiatrists to be mutually willing to change, agree on communication objectives and tools to improve the shared patient care (Gallais 2014). This has been shown to be facilitated with inter-professional meetings that allow physicians to get to know each other better and develop a sound collaboration (Fredheim *et al.* 2011). Also, considering that both medical specialties lack awareness about existing guidelines and mutual understanding about correspondence needs, projects to provide structured correspondence would be helpful (Odelola and Jabbar 2017).

Furthermore, multidisciplinary conciliation meetings and professional training sessions involving GPs, psychiatrists, nurses, psychologists and social workers would promote communication in an established local network (Saint-Pierre *et al.* 2018). Also, gaining knowledge about psychiatry and general medicine during university internships has been proposed to improve mutual understanding (Fovet *et al.* 2014). This could be extended to further professional education and encouraging interdisciplinarity in clinical practice guideline updates would help to ensure mutual collaboration is applied in practice.

Furthermore, a partnership charter between GPs and psychiatrists has been proposed to involve GPs in prescribing medication. In this proposed charter, the psychiatrist proposes a treatment that the GP would prescribe and manage. This shared care may help to limit co-prescriptions and interactions, allows the GP to monitoring treatments suggested by the psychiatrist, and enables intervention without consent if a hospitalization occurs.

Lastly, in some cases, administrative issues associated with the patient pathway, such as a patient’s right to free access to a psychiatrist until the age of 26 during emergency psychiatric treatment because of family or social services may hinder GP correspondence. In which case, little or no referring information from a GP (Hérisson 2015).

### Strengths and weaknesses

In this study, while the response rates of psychiatrists (33%) and GPs (23%) are relatively high compared to other research in the region (Supper *et al.* 2011), there remains a potential for selection bias. In particular, the two questionnaires were independent, so the respondents (GPs or psychiatrists) might not have their usual correspondent among the other respondents. This limits the conclusions of the study, but others research that have tried to perform mirrored surveys, focused on specific patients have had very small population. (Cohen *et al.* 2021) In addition, the study was declarative and did not focus on patient cases. The absence of a detailed, case-specific approach, combined with the potential for recall bias, limits the accuracy of the data. Also, performing the same analysis in all departments in one geographic region made it possible to eliminate potential differences linked to local organizations and unfavourable demographics in certain regional departments.

Also, we proposed an online data collection system, which suited the younger population at ease with the internet and so provided a high level of data quality as opposed to the paper version in Sarthe, which had a lot of missing data, limiting sub-groups analyses for the entire region. Although the GP population was more male-dominated and younger compared to the region and France and the age and sex of psychiatrists differed from the region and the rest of France, there was no difference with regard to the activity type (Table 3). Anonymity was used to limit the impact of desirability bias, but it remains possible that some individuals may alter their responses based on what they believe to be more acceptable.

In this study, we cannot determine precisely the respondent rate among the GPs surveyed, as the rate of GPs who used email in 2014 was not known. The length and structure of the questionnaire may have caused frustration or fatigue, reducing the rate of respondents. The quality of the data may also be biased by the doctors’ interest in the subject: GPs who are not interested in mental health may not have taken the time to answer the questionnaire and may have different expectations from those of the respondents. Lastly, two years lapsed between the first study in 2014 and the last in 2016, and several years before the results were published. During this period, it is likely that new tools (Shared medical records, technology-enabled care coordination, telemedicine, secure messaging, etc.) and organizations would have improved communication between healthcare professionals (Bensoussan and Prébois 2021; Iorfino *et al.* 2021; Odier and Falcoff 2021; Parker *et al.* 2023). Unfortunately, at the same time, the mental health of the population is deteriorating, and this problem is exacerbated by a decline in medical demography, particularly in France (Bergeat *et al.* 2021; Chevance *et al.* 2020; GBD 2019 Mental Disorders Collaborators 2022). Communication between professionals may therefore have been affected, and further studies are needed to assess these changes.

Although this study was conducted prior to the COVID-19 pandemic, its findings remain relevant in the current context, as many of the communication challenges identified between GPs and psychiatrists have likely been exacerbated by the health crisis. Indeed, the pandemic has led to an increased workload for GPs, with a significant rise in mental health issues, making regular and effective communication between healthcare professionals even more crucial (Bergeat *et al.* 2021; Hazo *et al.* 2021). In addition, the health system’s response to the pandemic, including the accelerated adoption of telemedicine and digital communication tools, may have changed the ways in which correspondence is exchanged. However, integrating these new tools remains a challenge, particularly due to its complexity (Sebai and Manzani 2023). As highlighted by recent studies discussing the vulnerabilities of patients with mental disorders during the pandemic, the mental healthcare system itself faced significant challenges, including a shortage of resources and difficulties in adapting to the increased need for care, further underscoring the critical importance of communication between GPs and psychiatrists during this period (Chevance *et al.* 2020). It is therefore important to conduct future research to assess whether these new modes of communication have helped address the issues identified in our study, or if they have introduced new barriers. While changes have occurred since the data collection, the challenges outlined in this study – particularly the frequency and quality of correspondence – remain pertinent and require continued attention to improve mental healthcare coordination.

## Conclusion

Quantitative and qualitative improvement in physician correspondence is invaluable for psychiatrists and GPs to collaborate efficiently. Increasing the awareness of CNQSP recommendations approved by the HAS and mutual awareness would improve the quantity and quality of information exchange.

## Supporting information

Bouton et al. supplementary material Bouton et al. supplementary material

## Data Availability

The datasets used and/or analyzed during the current study available from the corresponding author on reasonable request.

## Supplementary material

To view supplementary material for this article, please visit https://doi.org/10.1017/S1463423625100492
