# Supplementary material for: Improving frequency and content of referral correspondence between general practitioners and psychiatrists: a cross-sectional descriptive study
Source: Prim Health Care Res Dev. 2025 Oct 24;26:e88. doi: 10.1017/S1463423625100492 (PMC12571929; doi:10.1017/S1463423625100492)
Supplement: Bouton et al. supplementary material [file S1463423625100492sup001.docx]

**General Practitioners' Questionnaire**

| **Socio-demographic Characteristics** | |
| --- | --- |
| Questions | Responses |
| Sex: | - Male - Female |
| Do you practise: | - Privately - At a hospital - In a mixed setting |
| Age: | - Under 45 years - Between 46 and 61 years - Over 61 years |
| When did you start practising? | - Before 2002 - Between 2002 and 2005 - After 2005 |
| When did you defend your thesis? | - Before 2002 - Between 2002 and 2005 - After 2005 |
| When did you start your 3rd cycle (Internship, intern stage, end of externship)? | - Before 2002 - Between 2002 and 2005 - After 2005 |
| Have you undertaken additional training in mental health management? | - No - Yes |
| If yes, which one? | - University Diploma or Inter-University Diploma - Continuing Professional Education - Continuing Professional Development |
| Specify the title of the additional training |  |
| **Frequency of Correspondence** | |
| How often do you write a letter to your psychiatric colleagues for the first consultation of a patient you have referred? | - Always - Often - Rarely - Never |
| How often do you write a letter to your psychiatric colleagues during the joint follow-up of a patient? | - Always - Often - Rarely - Never |
| **Content of the Letters** | |
| How often is the letter sent to the psychiatrist simply an introductory note for reimbursement purposes within the care pathway? | - Very often - Often - Rarely - Never |
| When you write a letter to your psychiatric colleagues, do you raise questions regarding: | - Diagnostic hypotheses - The medication management initiated - the type of follow-up - Psychotherapeutic and pharmacological follow-up - Social management (sick leave, disability, Long-Term Condition status, safeguarding measures, Disability Support Files (MDPH files)) - Current clinical elements - Identified symptoms - Symptom progression |
| When you write a letter to your psychiatric colleagues, do you mention: | - Somatic history (comorbidities) - Life context (family status, work) - Psychiatric history - Addictions - Allergies - Drug intolerances - Status of current care |
| When you write a letter to your psychiatric colleagues, do you include the status of current care, along with: | - Psychotropic treatment initiated - Psychotropic treatments already tried - Other ongoing treatments - The degree of the patient's acceptance of the proposals resulting from the consultation with the psychiatrist - Other |
| If "Other", please specify: |  |
| **Frequency of Letters Received from Psychiatrists** | |
| How often do you receive letters from your psychiatric colleagues? | - Very often - Often - Rarely - Never |
| How often do you receive a letter from your psychiatric colleagues during a first consultation? | - Very often - Often - Rarely - Never |
| How often do you receive a letter from your psychiatric colleagues once the diagnosis is made? | - Very often - Often - Rarely - Never |
| How often do you receive a letter from your psychiatric colleagues during follow-up after a clinical change or care modification? | - Very often - Often - Rarely - Never |
| How often do you receive a letter from your psychiatric colleagues at the end of psychiatric care? | - Very often - Often - Rarely - Never |
| **General Practitioner’s Observations on Psychiatric Letters** | |
| How often does the letter contain answers on the diagnostic hypothesis? | - Always - Often - Rarely - Never |
| How often does it contain answers regarding medication management? | - Always - Often - Rarely - Never |
| How often does it contain answers on the organization of follow-up? | - Always - Often - Rarely - Never |
| How often does it contain answers on the request for psychotherapeutic care? | - Always - Often - Rarely - Never |
| How often does it contain answers regarding social management? | - Always - Often - Rarely - Never |
| How often does it contain answers about your role in follow-up? | - Always - Often - Rarely - Never |
| How often does it contain information on immediate evolving risks? | - Always - Often - Rarely - Never |
| How often does it contain information on clinical psychiatric surveillance elements? | - Always - Often - Rarely - Never |
| How often does it contain information about important environmental factors to consider (work environment, marital context, substances, etc.)? | - Always - Often - Rarely - Never |
| How often does it contain information on the need for sick leave? | - Always - Often - Rarely - Never |
| How often does it contain information on the need for a Long-Term Condition protocol? | - Always - Often - Rarely - Never |
| How often does it contain information on a personalized care plan? | - Always - Often - Rarely - Never |
| How often does it contain information on the proposed pharmacological treatment? | - Always - Often - Rarely - Never |
| How often does it contain information on biomolecular or biological surveillance elements? | - Always - Often - Rarely - Never |
| How often does it contain information on the psychotherapeutic methods in place? | - Always - Often - Rarely - Never |
| What other expectations do you have regarding the content of this letter? |  |
| **General Practitioner’s Expectations Regarding Psychiatric Letters** | |
| Would you like to receive letters from your psychiatric colleagues? | - No - Yes |
| At which point in the care pathway would you like to receive a letter from the psychiatrist? | - From the first consultation - Once the diagnosis is confirmed - During follow-up after a clinical or care modification |
| In the psychiatrist's letter, would you like to receive information regarding: | - Diagnostic hypotheses - Medication management - The overall organization of follow-up - The need for psychotherapeutic care - Social management - The role of the general practitioner in follow-up - Evolving risks - Clinical surveillance elements - Environmental factors - The need for sick leave - The need for Long-Term Condition status - A personalized care plan - Proposed pharmacological treatments - Medication surveillance elements - Psychotherapeutic modalities - Other |
| If "Other", please specify: |  |
| **Conclusion** | |
| Are you familiar with the HAS recommendations from 2010 regarding correspondence between psychiatrists and general practitioners? | - No - Yes |
| If yes, do they seem realistic to you? | - No - Yes |
| If no, why? |  |
| Evaluate the importance you place on managing mental health disorders and psychological suffering on the following scale: | - 0/1/2/3/4/5/6/7/8/9/10 |

**Psychiatrists' Questionnaire**

| **Socio-demographic Characteristics** | |
| --- | --- |
| Questions | Responses |
| Sex: | - Male - Female |
| Do you practise: | - Privately - At a hospital - In a mixed setting |
| Age: | - Under 45 years - Between 46 and 61 years - Over 61 years |
| When did you start practising? | - Before 2002 - Between 2002 and 2005 - After 2005 |
| When did you defend your thesis? | - Before 2002 - Between 2002 and 2005 - After 2005 |
| When did you start your 3rd cycle (Internship, intern stage, end of externship)? | - Before 2002 - Between 2002 and 2005 - After 2005 |
| Have you undertaken additional training in mental health management? | - No - Yes |
| If yes, which one? |  |
| **Frequency of Correspondence** | |
| How often do you write a letter to your general practitioner colleagues for the first consultation of a patient referred to you? | - Always - Often - Rarely - Never |
| How often do you write a letter to your general practitioner colleagues once the diagnosis is confirmed? | - Always - Often - Rarely - Never |
| How often do you write a letter to your general practitioner colleagues during follow-up after a clinical change or care modification? | - Always - Often - Rarely - Never |
| . How often do you write a letter to your general practitioner colleagues at the end of follow-up? | - Always - Often - Rarely - Never |
| **Content of the Letters** | |
| When you write a letter to your general practitioner colleagues, do you include information regarding: | - Diagnostic hypotheses - Medication management initiated by the general practitioner - Medication follow-up - Psychotherapeutic care - Current clinical elements - Follow-up clinical elements - Important environmental factors to consider - The need for sick leave - The need for Long-Term Condition status - Evolving risks |
| When you write a letter to your general practitioner colleagues, do you remind them of: | - Somatic history (comorbidities) - Life context (family status, work) - Psychiatric history - Allergies - Drug intolerances - Biomedical and biological elements |
| When you write a letter to your general practitioner colleagues, do you include the status of current care following the consultation, along with: | - A situational diagnosis - A therapeutic proposal - The contents of a prescribed prescription - A care pathway proposal - A psychotherapeutic care proposal - A social care proposal - A proposed distribution of roles in follow-up - The degree of the patient's acceptance regarding the proposals resulting from the consultation - Other |
| If "Other", please specify: |  |
| **Frequency of Letters Received from General Practitioners** | |
| How often do you receive letters from general practitioners? | - Very often - Often - Rarely - Never |
| Would you like to receive them? | - No - Yes |
| **Psychiatrist's Observations on General Practitioners' Letters** | |
| How often is the letter received from the general practitioner simply an introductory note for reimbursement purposes within the care pathway? | - Very often - Often - Rarely - Never |
| How often does it contain detailed reasons for the request? | - Always - Often - Rarely - Never |
| How often does it contain questions regarding diagnostic hypotheses? | - Always - Often - Rarely - Never |
| How often does it contain questions regarding medication management initiated? | - Always - Often - Rarely - Never |
| How often does it contain questions on the type of follow-up? | - Always - Often - Rarely - Never |
| How often does it contain a question about the request for exclusive psychotherapeutic care? | - Always - Often - Rarely - Never |
| How often does it contain questions regarding social care? | - Always - Often - Rarely - Never |
| How often does it contain information on identified symptoms? | - Always - Often - Rarely - Never |
| How often does it contain information on symptom progression? | - Always - Often - Rarely - Never |
| How often does it contain information on somatic history? | - Always - Often - Rarely - Never |
| How often does it contain information on psychiatric history? | - Always - Often - Rarely - Never |
| How often does it contain information on life context (family, work)? | - Always - Often - Rarely - Never |
| How often does it contain information on allergies? | - Always - Often - Rarely - Never |
| How often does it contain information on drug intolerances? | - Always - Often - Rarely - Never |
| How often does it contain information on addictions? | - Always - Often - Rarely - Never |
| How often does it contain information on the status of current care? | - Always - Often - Rarely - Never |
| How often does it contain information on current psychotropic treatments? | - Always - Often - Rarely - Never |
| How often does it contain information on previous psychotropic treatments? | - Always - Often - Rarely - Never |
| How often does it contain information on other treatments? | - Always - Often - Rarely - Never |
| How often does it contain information on the degree of the patient's acceptance regarding this consultation with the psychiatrist? | - Always - Often - Rarely - Never |
| What other expectations do you have regarding the content of this letter? |  |
| **Psychiatrist’s Expectations Regarding General Practitioners' Letters** | |
| In the general practitioner's letter, would you like to receive information regarding: | - Detailed reasons for the request - Diagnostic hypotheses - Medication management initiated - Type of follow-up request - Request for exclusive psychotherapeutic care - Initially identified symptoms - Symptom progression since initial care - Somatic history (comorbidities) - Psychiatric history - Life context - Allergies - Drug intolerances - Addictions - Current care status - Current psychotropic treatments - Previous psychotropic treatments - Other treatments - The degree of the patient's acceptance regarding the consultation - Other |
| If "Other", please specify: |  |
| Are you familiar with the HAS recommendations from 2010 regarding correspondence between psychiatrists and general practitioners? | - No - Yes |
| If yes, do they seem realistic to you? | - No - Yes |
| If no, why? | - No - Yes |
